# Rapid Visuomotor Responses Reflect Value-Based Decisions

**DOI:** 10.1523/JNEUROSCI.1934-18.2019

**Published:** 2019-05-15

**Authors:** Timothy J. Carroll, Daniel McNamee, James N. Ingram, Daniel M. Wolpert

**Affiliations:** ^1^Centre for Sensorimotor Performance, School of Human Movement and Nutrition Sciences, The University of Queensland, Brisbane 4072, Queensland, Australia,; ^2^Computational and Biological Learning Laboratory, Department of Engineering, University of Cambridge, Cambridge CB2 1PZ, United Kingdom, and; ^3^Zuckerman Mind Brain Behavior Institute, Department of Neuroscience, Columbia University, New York, New York

**Keywords:** decision-making, involuntary movement, reflex, value-based choice

## Abstract

Cognitive decision-making is known to be sensitive to the values of potential options, which are the probability and size of rewards associated with different choices. Here, we examine whether rapid motor responses to perturbations of visual feedback about movement, which mediate low-level and involuntary feedback control loops, reflect computations associated with high-level value-based decision-making. In three experiments involving human participants, we varied the value associated with different potential targets for reaching movements by controlling the distributions of rewards across the targets (Experiment 1), the probability with which each target could be specified (Experiment 2), or both (Experiment 3). We found that the size of rapid and involuntary feedback responses to movement perturbations was strongly influenced by the relative value between targets. A statistical model of relative value that includes a term for risk sensitivity provided the best fit to the visuomotor response data, illustrating that feedback control policies are biased to favor more frequent task success at the expense of the overall extrinsic reward accumulated through movement. Importantly however, the regulation of rapid feedback responses was associated with successful pursuit of high-value task outcomes. This implies that when we move, the brain specifies a set of feedback control gains that enable low-level motor areas not only to generate efficient and accurate movement, but also to rapidly and adaptively respond to evolving sensory information in a manner consistent with value-based decision-making.

**SIGNIFICANCE STATEMENT** Current theories of sensorimotor control suggest that, rather than selecting and planning the details of movements in advance, the role of the brain is to set time-varying feedback gains that continuously transform sensory information into motor commands by feedback control. Here, we examine whether the fastest motor responses to perturbations of movement, which mediate low-level and involuntary feedback control loops (i.e., reflexes), reflect computations associated with high-level, value-based decision-making. We find that rapid feedback responses during reaching reflect the relative probabilities and rewards associated with target options. This suggests that low-order components of the sensorimotor control hierarchy, which generate rapid and automatic responses, can continuously evaluate evolving sensory evidence and initiate responses according to the prospect of reward.

## Introduction

Every decision that an animal makes must ultimately be implemented through movement. Therefore, rational decision-making should take into account the properties and state of the motor system when weighing the desirability of options. Indeed, value-based decisions are sensitive to the physical costs of action (Croxson et al., 2009; Skvortsova et al., 2014; Manohar et al., 2015; Klein-Flügge et al., 2016; Shadmehr et al., 2016), and decisions about which action to perform can be rapidly adapted if the state of the body unexpectedly changes (Nashed et al., 2012, 2014). There is both neurophysiological and behavioral evidence that the state of the motor system reflects decision variables before a final commitment to act (Platt and Glimcher, 1999; Song and Nakayama, 2008; Resulaj et al., 2009; Pastor-Bernier and Cisek, 2011; Selen et al., 2012). For example, the firing rates of neurons in the dorsal premotor cortex reflect the reward associated with a potential target in their receptive field relative to alternative targets (Pastor-Bernier and Cisek, 2011). Similarly, the gains of long latency stretch reflexes track evidence accumulation when a perceptual decision must be reported by a motor response to a change in limb position (Selen et al., 2012), and movements initiated under uncertainty are strongly biased by factors that are critical to evaluating the expected relative values of alternative choices, such as their associated rewards and costs (Seydell et al., 2008; Landy et al., 2012; Schütz et al., 2012).

Although these observations confirm a tight coupling between decision-making and sensorimotor control, questions remain regarding the nature of the interactions between these processes. In particular, it is unclear to what extent decision variables modulate feedback control systems that are integral to effective movement. Our movements rely on flexible and hierarchical feedback control that effectively deals with noise and delays in sensory feedback by taking account of efference copy information. The selection of an “action” to be taken is therefore better conceived of as the specification of a feedback control policy that continuously transforms internal neural states and sensory inputs into motor outputs (Todorov and Jordan, 2002; Scott, 2004; Todorov, 2004). Such policies specify the gain of feedback loops at multiple levels of the sensorimotor hierarchy, such that all but the shortest latency spinal reflex arcs can be flexibly customized to the task context (Scott, 2016). The assessment of rapid feedback responses to sudden changes in the state of the body or the environment therefore provides a window into the computations upon which feedback control policies are based. Here we use this approach to ask whether feedback control systems are influenced by the relative value of potential reach goals. If such decision variables are to effectively shape motor behavior, their influence should be incorporated into feedback control policies and thus be observable in rapid feedback responses to sudden perturbations. This would require that some aspects of value-based “decision-making,” typically conceived of as a high-order cognitive computation, be implemented in the low-order components of the sensorimotor control hierarchy that generate rapid and automatic responses.

Here we examine whether rapid feedback responses to displacements of visual feedback of hand position are tuned to the relative values of alternative reach targets. Because the value of an option is the product of its contingent reward magnitude and probability, we first conducted separate experiments to determine whether rapid feedback responses are tuned to bring the hand closer to targets that carry greater rewards and to targets presented more frequently than the alternatives. As both components of value modulated feedback responses, we conducted a third experiment involving different combinations of reward and probability, to test how well statistical models of reward, probability, and relative value explain feedback response modulation. A relative value model with a term for risk sensitivity best fit the data, implying that low-level sensorimotor circuits can flexibly evaluate sensory information and rapidly tune motor responses according to the prospect of reward.

## Materials and Methods

### 

#### 

##### Participants.

Thirty-four self-reported, right-handed participants, without a reported neurological condition and with normal or corrected-to-normal vision, took part in one of three experiments (Experiment 1: 7 females, 5 males; age range, 18–32 years; Experiment 2: 5 females, 7 males; age range, 18–33 years; Experiment 3: 5 females, 5 males; age range, 21–37 years). All participants were naive to the purpose of the experiment. They received payment of between £20 and £35 with the amount depending on both experimental duration (1.5–2.5 h) and performance (Experiments 1 and 3; see below). The Cambridge Psychology Research Ethics Committee approved the experimental procedures, which conformed to the Declaration of Helsinki. All subjects gave written informed consent.

##### Experimental apparatus.

Participants made reaching movements while grasping the handle of a robotic manipulandum (vBOT) that constrained motion of the hand to the horizontal plane. The vBOT is a custom-built robotic device that measures the position of the handle and generates state-dependent forces at the handle end point (Howard et al., 2009). A six-axis force transducer (ATI Nano 25; ATI Industrial Automation) measured the forces applied by the subject at the handle. Hand position was measured by optical encoders (58SA, Industrial Encoders Direct). Visual feedback was provided using a computer monitor (ASUS, VG278H, 120 Hz) mounted above the vBOT and was projected veridically to the subject via a mirror. Subjects were prevented from viewing their hand directly, and the virtual reality system was used to overlay images such as targets and a hand cursor (0.5-cm-radius disk) in the plane of movement. Hand position and forces were sampled at 1000 Hz. The delay between position sampling and cursor display was measured with a photodiode as 24 ms at the center of the screen, and all reported response times are corrected for the display latency.

##### Task details.

**Figure 1.**
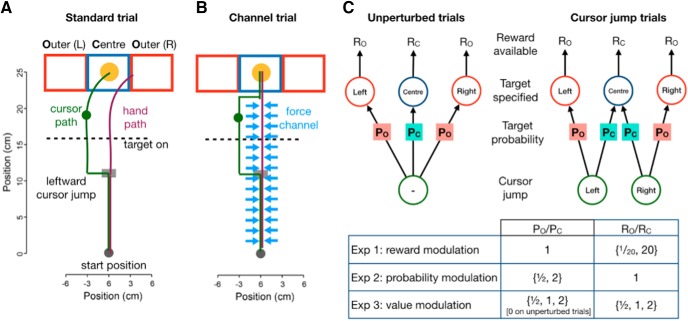
Schematic of the task and conditions. ***A***, Schematic of cursor and hand paths on a standard trial. Participants moved the cursor through a gray bar located 11 cm from the start position, at which point the cursor could jump either to the left (shown) or right or remain unperturbed. When the hand was 15.5 cm (or 16 cm; see Materials and Methods) from the start, a yellow target was displayed in one of the three boxes according to probabilities shown in ***C***. On cursor jump trials, participants had to move their hand in the opposite direction to the jump to acquire the center target, but in the same direction to acquire an outer target (case not shown). ***B***, Schematic for a channel trial in which the hand was constrained to a straight line to the center target by a force channel (blue arrows) generated by the robot. On cursor jump trials, the cursor was aligned to the final target when it was 3 cm from the target. On these trials, we measured the lateral force produced against the walls of the channel. ***C***, Manipulation of reward and target probabilities on unperturbed (left) and cursor jump (right) trials. In different experiments we manipulated the relative rewards (*R_O_*/*R_C_*) and target probabilities (*P_O_*/*P_C_*) associated with the outer and central targets. In the reward modulation experiment (Experiment 1) on unperturbed trials, all targets were possible with each target equally likely. On cursor jump trials, the center and outer target corresponding to the jump were equally likely. On all trials, the relative reward of the outer and central target had a 20-fold difference. In the probability modulation experiment (Experiment 2), all targets had the same reward but the relative probability of an outer to central target had a twofold difference. In the value modulation experiment (Experiment 3), both the reward and probability were manipulated on cursor jump trials (note that we did not include the condition in which relative reward and probability were both 1). In Experiment 3, on unperturbed trials only the center target was cued.

Each trial began with the participant's hand within a 0.55-cm-radius home circle, which was aligned with the body midline. Participants made a 25 cm reaching movement toward an array of three colored boxes (each 6 × 5 cm; Fig. 1*A*). Participants were asked to fixate a small gray cross that was displayed in the central box for ∼500 ms. The disappearance of the fixation cross was the go signal for the participant to initiate a reach toward the target array. If the hand speed did not exceed 10 cm/s within 550 ms of the go signal, an error message was displayed (“Too late”) and the trial was repeated. The low contrast of the dark gray cross on a black background made it difficult to perceive the go signal without fixation. To ensure the initial kinematics of movement were similar, participants were required to move the cursor through a small gray bar (2 cm wide × 1 cm long) located 11 cm from the home circle directly toward the center of the array. If the cursor did not pass through the intermediate gray bar, the screen turned red, all task information disappeared, and an error message was displayed (“Missed half-way target”). If the hand speed exceeded 60 cm/s before reaching the intermediate gray bar, the red screen appeared and another error message was displayed (“Too fast”). Trials aborted due to kinematic errors at the intermediate marker were repeated immediately.

On some trials, the cursor position was jumped laterally (left or right) by 3 cm when it reached the gray bar (the cursor was displayed on top of the bar). Critically, at this point, the final reach target was not yet specified. The final target was displayed as a yellow circle of 1.25 cm radius when the hand reached 15.5 or 16 cm (see below) from the home position. The participant was required to bring the cursor within the target for at least 50 ms within 800 or 850 ms (see below) of movement initiation. Feedback of whether the trial was successful and the amount of reward (if relevant, see below) were provided if the target was reached in time. If the target was not reached within the time limit, the cursor was extinguished and an error message was displayed (see below for details), but the trial was not repeated. In combination with the hand speed limit at the gray bar, the time limit served to constrain the peak speed of reaches to ∼50 cm/s (mean ± SD, 50.8 ± 2.5 cm/s).

There were two types of trials for both the unperturbed and cursor jump conditions. In standard trials, no forces were applied by the robot to the handle during the reach. Thus, participants had to correct any cursor displacement by moving the hand laterally to reach the target (Fig. 1*A*). On channel trials, the robot constrained the handle to move along a straight path from the home position to the center target (Fig. 1*B*) via a simulated stiff spring and damper (6000 N/m, 100 N/m/s). On all channel trials in which the cursor jumped, the cursor was aligned to the selected target when it was 3 cm from the target. Therefore, when the central target was specified, the cursor was realigned with the hand. In these trials, we measured the lateral forces that participants exerted against the walls of the channel in response to the cursor jump as a measure of the reflex gain (Franklin and Wolpert, 2008; Franklin et al., 2016).

##### Experiment 1: reward manipulation.

The first experiment was designed to determine whether the magnitude of the earliest visuomotor response to a cursor jump is sensitive to the reward associated with potential targets, even before a target is specified. Participants completed blocks of 102 trials. Each block consisted of the following trials: (1) 18 context trials with 6 standard unperturbed reaches to each of the three targets, which were performed first to illustrate the reward associated with each target in that block and were not analyzed; (2) 36 unperturbed trials with 12 reaches to each target, which were standard except for 6 channel trials to the central target; and (3) 48 cursor jump trials with 24 left cursor jump trials with an equal number of left and central targets and 24 right cursor jump trials with an equal number of right and central targets. All trials were standard except for six channel trials to the central target for both cursor jump directions.

After the 18 context trials (trial 1) that were performed at the start of each block, all remaining trials (trials 2 and 3) were performed in a random order. Participants completed four familiarization and six experimental blocks. In the familiarization blocks, task success was signaled only by the display of the text “Hit” or “Miss” immediately after each trial, and the color of the boxes surrounding the target array was varied randomly. The familiarization blocks allowed participants to learn the task and were also used to identify the appropriate time constraint for target acquisition (800 or 850 ms) and the hand position at which the target was revealed (15.5 or 16 cm). The goal was to make the task difficult for each participant, as pilot data showed considerable interindividual differences in performance, and that reward-related effects were minimal if subjects could reach the target on every trial. We chose a setting for movement duration and position at which a target was specified for the remaining blocks to obtain a hit rate of 70–85% (across all three experiments, the mean ± SD hit rate on standard trials was 73.8 ± 9.8%).

In the experimental blocks, rewards associated with potential targets were signaled by the colors of the three boxes. Targets that appeared inside boxes of one color (red or blue, counterbalanced across subjects) were associated with high reward (£10), whereas targets that appeared inside a box of the other color were associated with low reward (50p). In six alternating blocks, either the two outer targets were worth £10 and the center target worth 50p, or vice versa (Fig. 1*C*, reward ratios of 1/20 or 20). If a high reward target was hit, an image of a £10 note was displayed on the screen and a pleasant “ding” sound was played. If a low-reward target was hit, an image of a 50p coin was displayed and no sound was played. If the target was missed, the text “Too late—no bonus” was displayed. Subjects were informed that one trial in which each reward type had been available would be selected at random at the conclusion of the experiment. If the trial of that type was hit, then they would receive the associated reward (£10 or 50p), whereas if the trial was missed, no reward would be given. All participants were given £20 to compensate them for their time, so the final payment made to each person could be £20, £20.50, £30, or £30.50.

A final 11th block was included to measure the size and latency of the rapid visuomotor response to cursor jumps in the absence of target uncertainty. The block involved reaches to a single, central target that was visible throughout each trial. The first 12 trials were standard unperturbed trials and were not analyzed, and the remaining 90 trials were composed of 30 standard unperturbed trials, 12 standard trials each with left and right cursor jumps, 12 unperturbed channel trials, and 12 channel trials each with left and right cursor jumps. The reward associated with all trials was 50p.

##### Experiment 2: probability manipulation.

The second experiment was designed to determine whether the magnitude of the earliest visuomotor response to a cursor jump is sensitive to the probability with which potential targets appear. In this experiment, no financial rewards were contingent upon task performance. Participants first completed four familiarization blocks that were identical to those in Experiment 1, followed by six blocks in which either the center target or the two outer targets appeared more frequently, and one final block that corresponded to the last block of Experiment 1, in which a single target was visible throughout the reach. As above, these familiarization trials were used to keep target hit rates at ∼70–85% in the remainder of the experiment.

In the six blocks with probability manipulation, the probability of each target being presented was signaled by the color of the box surrounding each potential target location. Targets inside boxes of one color (red or blue, counterbalanced across subjects) were presented twice as often as those inside boxes of the other color. That is, the probability of left/center/right targets was either 0.4:0.2:0.4 or 0.25:0.50:0.25. When a target was hit, the text “hit” was displayed on the screen and a pleasant “ding” sound was played. If the target was missed, the text “Miss” was displayed and no sound was played. All participants were given £20 if the experiment lasted up to 2 h, plus £2.50 for any additional periods of 15 min.

Each block consisted of the following: (1) 12 unperturbed context trials with targets presented exclusively within colored boxes defining the more likely location (in alternating blocks: either in the center or at the two outer locations), and these trials were performed first, were included to illustrate the location of the more probable targets, and were not analyzed; and (2) the number of remaining trials in each block depended on whether the center or outer targets were presented more frequently in that block. In all cases, half the trials to the center target were channel trials.

If the center target was more probable, the remaining 100 trials included 40 unperturbed trials (target numbers 10:20:10) and 60 cursor jump trials with (10:20:0) for left cursor jumps and (0:20:10) for right cursor jumps.

If the outer targets were more probable, the remaining 110 trials included 50 unperturbed trials (20:10:20) and 60 cursor jump trials with (20:10:0) for left cursor jumps and (0:10:20) for right cursor jumps.

##### Experiment 3: value manipulation.

The third experiment included manipulations of both reward and probability to determine whether the magnitude of the earliest visuomotor response to a cursor jump scales with the relative value of potential targets. The first block of 102 trials was identical to the single trials blocks of Experiments 1 and 2.

Participants then completed two familiarization blocks of 120 standard trials consisting of 24 trials each of (1) unperturbed, (2) left and (3) right cursor jumps to the center target, and (4) left and (5) right cursor jumps to the left and right targets, respectively. As above, these familiarization trials were used to keep target hit rates at ∼70–85% in the remainder of the experiment. A key difference in this design compared with the first two experiments is that we included channels on trials in which the outer target was specified after a cursor jump. On these trials, the cursor was jumped again to align with the specified (outer) target when it was 3 cm from the target.

Participants performed eight experimental blocks in which the relative probability and relative rewards were varied. After a cursor jump, the relative probability (*P_O_*/*P_C_*) of the outer versus center target being specified was either 0.5, 1, or 2, and the relative reward for the outer versus center target (*R_O_*/*R_C_*) was either 0.5, 1, or 2. We examined all combinations (Fig. 1*C*) of relative reward and probability in different blocks (except where both were 1). Blocks were performed in pairs with *R_O_*/*R_C_* = *r* and *P_O_*/*P_C_* = *p* for one block followed by *R_O_*/*R_C_* = 1/*r* and *P_O_*/*P_C_* = 1/*p* for the other. The pairs were performed in a pseudorandom order.

The first 18–20 trials of each block were standard trials that served to illustrate the reward and probability characteristics of the block. These initial trials were not analyzed. The remaining 118 trials involved 108 cursor jump trials and 10 unperturbed trials, randomly intermixed. The center target was cued on all the unperturbed trials; 5 were channel trials and 5 were standard trials. The 108 cursor jump trials comprised 72 standard trials and 36 channel trials. Half of these involved a left cursor jump, and half involved a right cursor jump. The number of trials in which the center and outer targets were cued on these cursor jump trials is defined by the relative probabilities shown in Figure 1*C*. The center target was cued twice as often as the outer target for each direction of cursor jump (*P_O_*/*P_C_* = 0.5), cued half as often as the outer target for each cursor jump direction (*P_O_*/*P_C_* = 2), or cued the same number of times for each cursor jump direction (*P_O_*/*P_C_* = 1). Due to a coding error, one trial in each block of trials was assigned to an incorrect condition (with respect to the probabilities defined above). This resulted in an average deviation from the intended probability ratios of 0.19 ± 0.56% (group mean ± SD).

As in Experiment 1, rewards associated with potential targets were signaled by the colors of the three boxes surrounding the potential target array. Boxes of one color (red or blue, counterbalanced across subjects) were associated with high reward (£10), and boxes of the other color were associated with low reward (£5). If a high-reward target were hit, a large image of a chest of gold and the text “£10” were displayed on the screen and a pleasant “ding” sound was played. If a low-reward target were hit, a (50%) smaller image of chest of gold was displayed, the text “£5” was displayed, and a brief, medium-pitched tone was played. If the target was missed, the text “Too late—no bonus” was displayed. Subjects were informed that one trial in which each reward type had been available would be selected at random at the conclusion of the experiment. If the trial of each type had been hit, then they would receive the associated reward (£10 or £5), whereas if the trial was missed, no reward would be given. All participants were given £20 to compensate them for their time, plus £2.50 for any additional periods of 15 min, so the final payment made to each person could be £20, £25, £30, or £35 in addition to extra time payments.

##### Experimental design and statistical analysis.

The velocity and force (on channel trials) from each trial were low-pass filtered at 50 Hz, and the filtered velocity differentiated to provide end point acceleration. The average lateral force time series exerted by each subject during unperturbed channel trials was subtracted from forces exerted on each channel trial in which there was a cursor jump. Similarly, the average acceleration time series recorded during unperturbed standard trials were subtracted from standard cursor jump trials. The primary measure of the short latency response to a visuomotor displacement was the average (subtracted) lateral force exerted against the walls of the force channel between 170 and 220 ms after the cursor was displaced. We also calculated the average lateral acceleration of the hand in the same time window after cursor displacement in standard trials.

The decision to use of an analysis window of 170–220 ms was taken to make our analysis comparable to the majority of recent articles that used the cursor jump paradigm (Dimitriou et al., 2013; Franklin et al., 2014, 2016; Gallivan et al., 2016). Note that voluntary responses to cursor jumps occur ∼320 ms after the perturbation (Franklin and Wolpert, 2008), and voluntary responses to target jumps occur ∼220 ms after the perturbation (Day and Lyon, 2000). However, it is also of interest to determine the onset time at which any effect of target value becomes evident. To this end, we used signal detection theory on a subject by subject basis to identify the time at which the force responses to cursor jumps first diverged between blocks in which the outer and center targets were more valuable. This method was based on the one used by Weiler et al. (2015) with two modifications. We generated a receiver operating characteristic (ROC) curve for every 1 ms sample and calculated the area under the ROC (aROC) curves for the ability to distinguish between the responses for which outer and center targets were more valuable. As we are interested in the time point at which this difference emerges in the force responses, we examine the time point where the information begins to deviate from chance. To do this, we excluded aROC after the point when the aROC exceeded 0.62 for three consecutive samples. This is reduced from the values of 0.75 used in the study by Weiler et al. (2015) as the onset of the target (which occurred after that hand had moved a further 4.5 cm from the cursor jump location, i.e., ∼100 ms later) affected the movement so that some curves did not reach a value of 0.75. We then fit a dog-leg to the aROC data (flat line at aROC of 0.5 followed by a linear component). In addition, given the lower aROC criterion, to be conservative in our estimate of the divergence time, we took the later of the two times as the onset time of the response: (1) the end of the flat portion of the fit; and (2) the last local minimum in the aROC curve. This method provides a sensitive and conservative measure of the first time at which the visuomotor responses are modulated by expected value. We compared this time with that of the first force divergence between cursor jump and unperturbed trials in the single-target experiment block to establish whether the modulation of value-based response magnitude is evident from the earliest component of the visuomotor response.

In Experiments 1 and 2, the central target was selected on all channel trials, and we only analyzed corresponding standard trials in which the center target was presented. Group effects were analyzed with two-way repeated-measures ANOVA [three block types (central target more rewarded/probable, outer targets more rewarded/probable, single-target control trial) × 2 cursor jump directions (left, right)]. Effect sizes are reported as partial η^2^ statistics for relevant main and interaction effects. Greenhouse–Geisser corrections were applied to the degrees of freedom where violations of the assumption of sphericity were detected, and Holm–Bonferroni *post hoc* contrasts were used to assess pairwise differences between means. Statistica version 13.2 (Dell Software) was used for these tests.

Experiment 3 differed in that some reaches were channel trials even though an outer target was presented. To confirm that the eventual location of the target did not affect the early force response to cursor jumps within our analysis window of 170–220 ms, we again used signal detection theory to provide a sensitive measure of the first time at which the force response to central and outer targets began to diverge after a cursor jump. Note that the target was not revealed until the hand had moved a further 4.5–5 cm (∼100 ms) beyond the point at which the cursor jump was applied. We used the ROC approach described above to distinguish between trials in which outer and center targets were presented, and found that force response diverged after ∼230 ms (mean ± SD initiation time: left cursor jumps, 233 ± 15 ms; right cursor jumps, 228 ± 13 ms).

##### Hierarchical Bayesian modeling.

To test the extent to which early responses to visual perturbation vary systematically with the reward, the probability, and value of potential targets in Experiment 3, we performed hierarchical Bayesian modeling using custom Python scripts. Specifically, we fitted a hierarchical Bayesian model of group-level and subject-specific parameters in our models of response gains (Kruschke, 2010). These parameters were the slopes and intercepts linearly relating log value, probability, or reward to gains as well as the weighting parameter in the risk-sensitive model. Analyzing the data in this manner facilitates the identification of heterogeneous probability, reward, and risk attitudes across the population (as captured by the subject-level parameters) while making parameter estimation more robust to noise in response gains by partially pooling variance across subjects (due to the use of a prior over group-level variance). At the group-level, we specified weak priors for the mean (Gaussian, mean = 0, SD = 10000) and variance (half Cauchy, mean = 0, scale = 5) for all parameters. All priors were selected independent of the data, based on previously established defaults used in hierarchical modeling analysis. At the subject-level, parameters for each subject were modeled separately using Gaussian (probability, reward, and value parameters) or bounded Gaussian (risk-weighting parameter) distributions with means and variances drawn from the estimated group-level distributions. Error variance prior was modeled using a half Cauchy distribution (scale = 5). Parameters were fitted by maximum a posteriori estimation. The posterior was approximately inferred using Markov Chain Monte Carlo sampling based on the No-U-Turn algorithm (Salvatier et al., 2016). The sampler was tuned using 1000 samples, and 30,000 samples were then drawn. After discarding the first 5000 samples, which, due to nonstationarity, are relatively unlikely to be representative of the posterior, the remaining 25,000 were used for parameter estimation. Chain convergence was established based on the R-hat diagnostic values (which were very close to 1) and visual inspection of the sample traces.

##### Code accessibility.

All code used for data analysis will be provided upon written request to the corresponding author.

## Results

### Experiment 1: reward manipulation

The first experiment was designed to determine whether the magnitude of rapid feedback responses to displacements of visual feedback of the hand location is sensitive to the rewards associated with equally probable reach targets. Participants grasped the handle of a robotic interface, and received visual feedback of the position of their hand via a cursor that was overlaid into the plane of movement. They were required to initiate reaches toward the center of a three-target array (Fig. 1*A*), and the target was specified from the three alternatives late in the movement. The three targets were associated with different rewards; either the central target was associated with 20 times (£10 vs 50p) greater reward than the two outer targets, or vice versa. On some trials (four of every seven trials), the cursor was displaced (i.e., jumped) 3 cm to the left (Fig. 1*A*, example) or right of the hand location just before the midpoint of the movement (102.9 ± 9.3 ms, mean ± SD, before the target was specified). The experiment was designed (Fig. 1*C*) so that on these trials the center target and the outer target, toward which the cursor jumped, had equal probability of being specified (and the opposite outer target was never specified). On trials in which the cursor did not jump, all three targets were equally likely. We also ran a control condition in which only a single central target was presented.

On standard trials the hand was unconstrained by the robotic interface (Fig. 1*A*) and participants could correct for cursor jumps. To assess the strength of the visuomotor response to such a perturbation, a force channel was used on a subset of the trials (channel trials; Fig. 1*B*) in which the center target was cued to constrain the hand to a straight-line path to the target. The strength of the response was then assessed as the mean lateral force exerted into the wall of the channel from 170 to 220 ms after the cursor jump was applied. A rapid, involuntary response is known to start at ∼150 ms for single-target trials, and the voluntary response occurs at ∼320 ms (Franklin and Wolpert, 2008). Therefore, our measure captures the earliest response to visual error that is automatic and not under conscious control.

**Figure 2.**
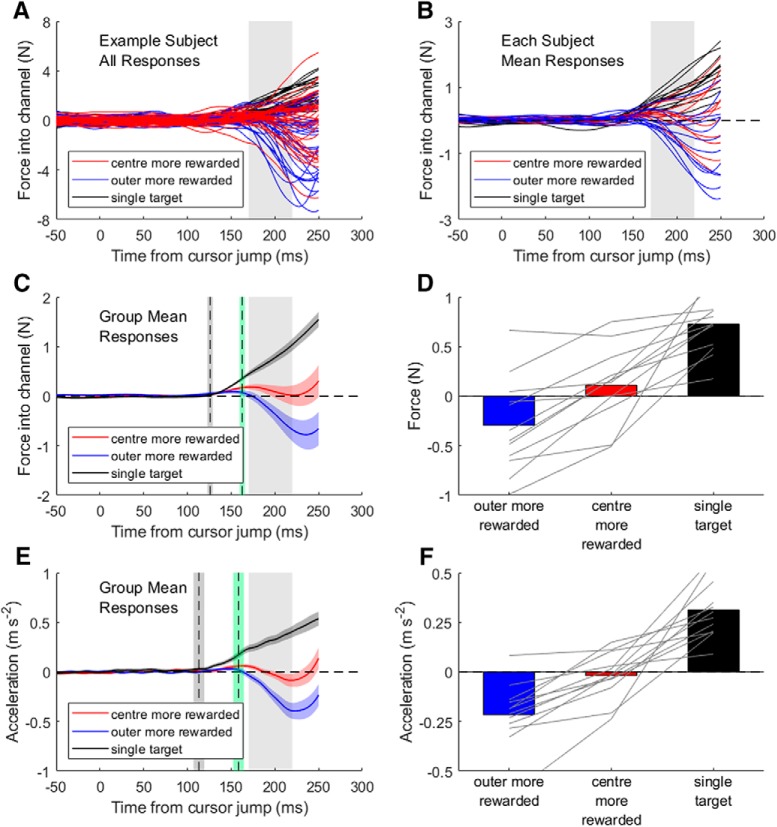
Force and acceleration responses to cursor jumps when different rewards were associated with center and outer targets. Note that the center target was ultimately cued on all trials shown here. Responses to left and right cursor jumps are pooled for all plots (right force signs are flipped), such that positive forces and accelerations represent a correction toward the center target. The gray highlighted zone indicates the period over which force and acceleration responses were averaged for analysis. ***A***, Force response traces on every individual trial in which there was a cursor jump from an example subject. ***B***, Average force responses across all trials for each participant; each trace is the average of all trials from center-rewarded, outer-rewarded, and single-target conditions completed by a different participant. ***C***, Group average ± SEM lateral forces exerted into the wall of the force channel. Vertical dashed lines and shading illustrate the mean ± SEM onset times of the visuomotor response in the single-target condition (gray) and the first time of response divergence between trial blocks with the outer versus center targets more probable (green). ***D***, Group averages (bars) and individual subject averages (gray lines) of the mean force exerted against the channel wall between 170 and 220 ms after the cursor jump. ***E***, Group average ± SEM hand acceleration traces on standard trials, when participants had to correct for the cursor jump to hit the target, as in Figure 1*A*. Vertical lines depict response onsets as per ***D***. ***F***, Corresponding hand acceleration group averages (bars) and individual subject averages (gray lines).

Figure 2 shows the lateral forces that participants exerted in the force channel just after the cursor jumped either left or right. The data for right and left cursor jumps have been combined by reversing the sign of the force for rightward cursor jumps, and the traces have been truncated at 250 ms to isolate the early latency response to the cursor jump. Figure 1*A* shows force traces from each individual trial in the three conditions for an example participant and illustrates that visuomotor responses to the cursor jump began at ∼125 ms in the control condition in which only the center target was rewarded (ROC analysis showed that perturbed force responses first deviated from force responses on no perturbation trials at 126 ± 11 ms, mean ± SD). Notably, some early responses were in the same direction as the cursor jump, especially in trials in which the outer targets were more rewarded. The effects of reward can be seen more clearly when average force traces for each participant are plotted in Figure 1*B*, and when the group averages (±SE) are plotted in Figure 1*C*. For the control condition (one block of 102 trials), in which only the central target was displayed (gray line), the trace shows a typical strong visuomotor response that is in the opposite direction to the cursor jump (positive force), thereby trying to return the hand to the central target. For the blocks in which all three targets were possible (six blocks of 102 trials, alternating between blocks in which the center and outer targets were rewarded more), the cursor jump moved the cursor closer to one of the outer targets and away from the center target. In such cases (red and blue), the average responses were weaker than in the single-target condition. This reflects the variability in response amplitude and direction observed across trials and participants when there was more than one potential target (Fig. 1*A*,*B*, blue and red traces). This may be because the ultimate target was uncertain, and if the final target happened to be the outer target toward which the cursor jumped, there would be less need to respond to the jump. Splitting the responses by whether the center (red trace) or outer (blue trace) targets were rewarded more shows that the responses are biased toward the more rewarded target.

Figure 2*D* shows the group results for the average lateral force exerted from 170 to 220 ms after the cursor jump. As reflected in the force–time plots, force responses that would tend to correct the cursor jump and bring the cursor back toward the center target were greatest for the single-target control condition. Strikingly, however, when the outer targets were more highly rewarded, the group average response was in the same direction as the cursor jump toward the outer target, that is, a reversal of the normal reflex response. The sign of the response was not uniform across participants, and not statistically different from zero at group level (single-sample *t* test, *t*_(11)_ = −2.2, *p* = 0.054). However, responses were significantly biased in the direction of the cursor jump for 7 of the 12 participants (*p* < 0.05; single-sample *t* tests against 0 for each subject, uncorrected for multiple comparisons), which shows that short latency responses to visuomotor error signal were reversed in some people. Moreover, the trend for corrective responses to be largest in single-target trials, followed by conditions in which the center and then the outer targets were more highly rewarded, was highly consistent across subjects. A repeated-measures ANOVA (3 target conditions × 2 cursor jump directions) gave a significant effect of condition (*F*_(1.3,14.2)_ = 29.4, *p* < 10^−4^, η^2^ = 0.73). There were no other significant main or interaction effects (both *p* > 0.20). Holm–Bonferroni *post hoc* contrasts showed that response size was significantly greater for the control condition than for either of the asymmetric reward conditions (both *p* < 10^−3^), and that the corrective force toward the center target was greater when the center target was more highly rewarded than the outer targets (*p* < 0.01).

An ROC analysis was used to identify the first time after the perturbation of visual feedback that the force responses differed for trials performed when the outer versus the center targets were more rewarded. The vertical dotted green line (mean divergence point with shaded ± SE) shows that the outer and inner rewarded trials began to diverge at 163 ms, which was significantly later than the onset of the response determined in single-target trials (paired *t* test, *t*_(11)_ = −3.3, *p* = 0.007). Thus, the effect of reward on the rapid feedback response was not reliably apparent until ∼35 ms after response onset. This timing discrepancy raises the possibility that there are multiple components to fast visuomotor responses to cursor jumps (i.e., perhaps analogous to the multiple components of long-latency stretch reflexes) and that the earliest response component is not affected by reward. To our knowledge, however, in no previous work has rapid visuomotor responses been dissociated into multiple components. Moreover, the early components of the responses are smallest in magnitude and therefore more subject to noise. We therefore interpret these timing effects cautiously at this stage and leave the resolution of this intriguing question for future work. Critically, even if there are multiple components to rapid visuomotor responses, the effect of reward was apparent within our analysis window of 170–220 ms. This is well before the onset of voluntary responses to cursor jumps (∼320 ms; Franklin and Wolpert, 2008) and lies within the standard epoch typically used to measure automatic and involuntary feedback responses. An analysis of the lateral acceleration on standard trials and in which either the outer target or the central target could have been cued showed a very similar separation of responses (Fig. 2*E*,*F*). To simplify comparison with the channel trials, we only analyzed the trials in which the center target was eventually cued. Again, there was a significant effect of condition (*F*_(1.2,12.9)_ = 38.3, *p* < 10^−4^, η^2^ = 0.78), and no other significant main or interaction effects (both *p* > 0.4). Holm–Bonferroni *post hoc* contrasts showed that response size was significantly greater for the control condition than for either of the asymmetric reward conditions (both *p* < 10^−4^), and that the corrective acceleration toward the center target was greater when the center target was more highly rewarded than the outer targets (*p* < 0.01). These data confirm that the tendency for rapid visuomotor responses to be biased toward high value targets is not specific to movements made in a force channel, but are also apparent when a change in the limb trajectory was required to correct the visual error.

To determine whether early, involuntary responses to a cursor jump were important for task performance, we also compared hit rates for targets that were associated with high and low rewards. We focused particularly on standard, unconstrained trials in which there was a cursor jump, when the participants were required to correct the cursor trajectory to hit a target. When pooled across center and outer targets, the hit rate was significantly greater for trials in which the cued target was associated with the high (77 ± 10%, mean ± SD), rather than low reward (69 ± 12%; paired *t* test, *t*_(11)_ = 2.9, *p* = 0.015). Thus, the bias in rapid feedback response toward more highly rewarded targets was associated with more successful acquisition of high-reward targets. This suggests that feedback control policies can be rapidly (i.e., in <100 trials over a few minutes) modified to increase the value of motor outcomes, in the absence of changes in the physical characteristics of the task.

### Experiment 2: probability manipulation

In the second experiment, there was no monetary reward associated with the targets, but we varied the relative probability of the central versus outer targets in separate blocks. The experiment was designed (Fig. 1*C*), so that on cursor jump trials within a block, the probability *P_C_* of the center target being specified was either half or twice the probability *P_O_* of the outer target (toward which the cursor jumped) being specified. Similarly, on trials in which the cursor did not jump, each outer target had either twice or half the probability of being presented compared with the center target. There were three blocks of 100 trials with *P_O_* < *P_C_*, which were alternated with three blocks of 110 trials with *P_C_* < *P_O_*.

**Figure 3.**
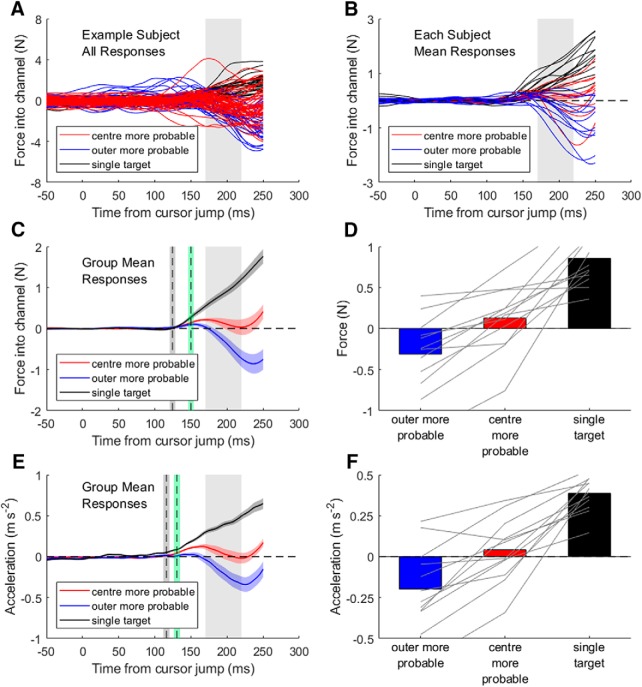
Force and acceleration responses to cursor jumps when center and outer targets had different probabilities of selection. Sign conventions are as for Figure 2. ***A***, Force response traces on every individual trial in which there was a cursor jump from an example subject. ***B***, Average force responses across all trials for each participant; each trace is the average of all trials from center more probable, outer more probable, and single-target conditions completed by a different participant. ***C***, Group average ± SEM lateral forces exerted into the wall of the force channel. Vertical dashed lines and shading illustrate the mean ± SEM onset time of the visuomotor response in the single-target condition (gray) and the first time of response divergence between trial blocks with the outer versus center targets more probable (green). ***D***, Group averages (bars) and individual subject averages (gray lines) of the mean force exerted against the channel wall between 170 and 220 ms after the cursor jump. ***E***, Group average ± SEM hand acceleration traces on standard trials, when participants had to correct for the cursor jump to hit the target. Vertical lines and shading as per ***D***. ***F***, Corresponding hand acceleration group averages (bars) and individual subject averages (gray lines).

Figures 3*A–C* show the force traces from these two conditions and from the single-target condition. The results are strikingly similar to those observed for reward manipulations. Again, a robust early response was seen in the single-target condition (gray) and the responses were smaller when there was target uncertainty (red and blue). Figure 3*C* also shows that the onset time of the response in the single-target condition, determined by ROC analysis, was 125 ms (Fig. 3*C*, dashed vertical black line with gray shading shows mean ± SE). When the outer targets were more probable, the cursor jump led to an average response that would have brought the hand closer to the outer target. In contrast, when the center target was more probable the response was weaker. The ROC analysis used to identify the first time at which the force responses differed for trials performed when the outer versus the center targets were more probable showed that trials obtained when the outer and inner targets were more probable trials began to diverge at 151 ms (Fig. 3*C*, vertical dashed green line with shading shows mean ± SE). As was the case for reward asymmetry revealed in Experiment 1, the effect of probability on visuomotor response magnitude was significantly delayed from the onset of the response in single-target trials (paired *t* test, *t*_(11)_ = −2.5, *p* = 0.03).

Figure 3*B* shows the group results for the average lateral force exerted from 170 to 220 ms after the cursor jump. As was the case for more highly rewarded targets, when the outer targets had a higher probability of being selected, the group average response was in the same direction as the cursor jump: toward the outer target. Here, the effect was statistically different from zero at group level (*t*_(11)_ = −2.3, *p* = 0.04), and responses were significantly biased in the direction of the cursor jump for 5 of the 12 participants (*p* < 0.05; single-sample *t* tests against 0 for each subject). Once again, the trend for corrective responses to be largest in single-target trials, followed by conditions in which the center and then the outer targets were more probable, was highly consistent across subjects (Fig. 3*B*). A repeated-measures ANOVA (3 target conditions × 2 cursor jump directions) gave a significant effect of condition (*F*_(1.3,14.2)_ = 38.7, *p* < 10^−5^, η^2^ = 0.78). There were no other significant main or interaction effects (both *p* > 0.2). Holm–Bonferroni *post hoc* contrasts showed that response size was significantly greater for the control condition than for either of the asymmetric probability conditions (both *p* < 10^−4^), and that the corrective force toward the center target was greater when the center target was more probable than the outer targets (*p* < 0.01).

An analysis of the lateral acceleration on standard trials showed a similar separation of responses (Fig. 3*C*,*D*). Again, there was significant effect of condition (*F*_(1.5,16.3)_ = 42.0, *p* < 10^−6^, η^2^ = 0.79), and no significant main effect of cursor jump direction (*F*_(1,11)_ = 0.17, *p* = 0.6). However, there was a significant interaction effect between the probability condition and cursor jump direction, such that greater response sizes were apparent for leftward than rightward cursor jumps (*F*_(1.2,13.7)_ = 5.7, *p* = 0.03, η^2^ = 0.34). Holm–Bonferroni *post hoc* contrasts showed that response size was significantly greater for the control condition than either of the asymmetric probability conditions regardless of cursor jump direction (all *p* < 10^−4^). Despite the significant interaction effect, the corrective acceleration toward the center target was significantly greater when it was more probable than the outer targets for both leftward cursor jumps (*p* < 10^−4^) and rightward cursor jumps (*p* = 0.02). This subtle lateral asymmetry presumably reflects biomechanical factors associated with the impedance of the limb, since the corresponding force channel responses were symmetric.

As was the case for the target reward manipulation, hit rates for targets that were more probable (82 ± 9%, mean ± SD) were significantly greater than for targets that were less probable (73 ± 14%; paired *t* test, *t*_(11)_ = 5.4, *p* = 2.2 × 10^−4^). Thus, rapid feedback response size was biased toward targets that were either more probable or more highly rewarded, and in both cases the modulation of automatic visuomotor responses was associated with more successful acquisition of high-value targets. Since the value of an option is determined both by reward magnitude and how often the reward is available, we designed a third experiment to determine whether these results based on independent reward and probability manipulations can be unified within the framework of value-based decision-making.

### Experiment 3: value manipulation

**Figure 4.**
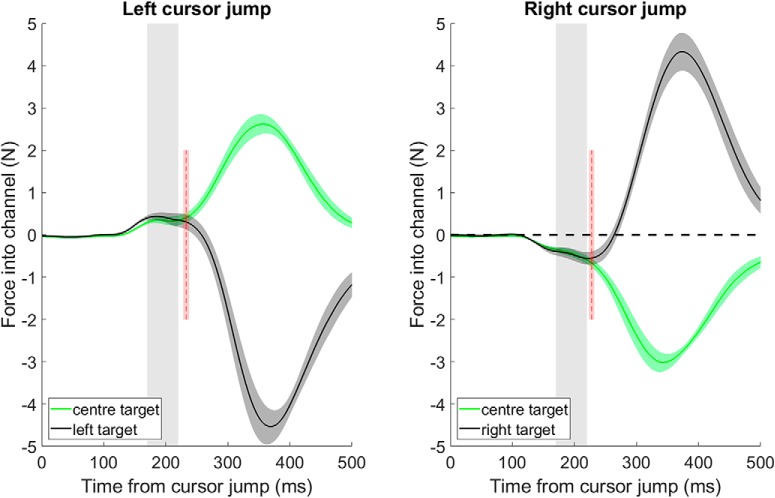
Force responses to left and right cursor jumps in Experiment 3 when the center and outer targets were ultimately specified. Group average ± SEM lateral forces exerted into the wall of the force channel show that the analysis window (170–220 ms after the cursor jump) was before the first point at which the force responses for center and outer targets diverged (red dotted line and shaded region represent the mean ± SEM of the point of first divergence as assessed by ROC analysis).

In a third experiment we varied both the relative reward and probability of specifying the center or outer target. The relative reward for the outer versus central target (*R_O_*/*R_C_*) was 0.5, 1, or 2 in separate blocks. After a cursor jump, the relative probability (*P_O_*/*P_C_* also fixed within a block) of the outer versus center target being specified was 0.5, 1, or 2 (Fig. 1*C*). We examined all combinations of relative reward and probability in different blocks (except where both were 1, and hence, eight blocks). These combinations led to five different relative values (i.e., the product of relative reward and probability) between the center and outer targets. In contrast to the first two experiments, we included channel trials in which the outer target was specified after a cursor jump. On these trials, the cursor jumped to the specified (outer) target at the end of the movement (i.e., at 22 cm, long after the response had been measured). We used ROC analysis (see Materials and Methods) to compare these trials, split by whether the center or the outer target was specified. This showed that the force response diverged on average 233 and 228 ms (for left and right targets) after the perturbation (Fig. 4). As this was outside our window for measuring the visuomotor response, we combined these trials in our analysis.

**Figure 5.**
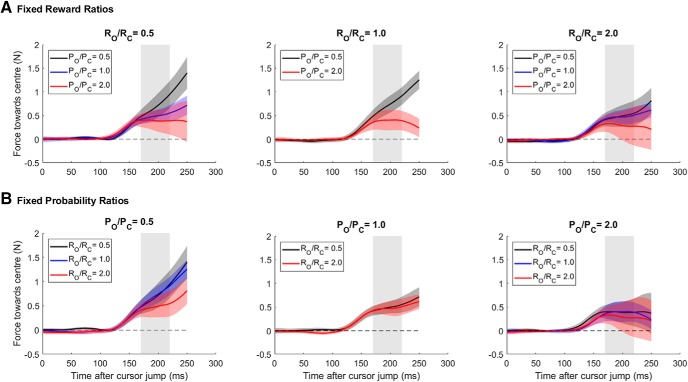
Force responses to cursor jumps for all eight conditions in which the probabilities and rewards associated with center and outer targets were varied. ***A***, Each panel shows data for trials with different reward ratios, with traces within a panel grouped by different target probability ratios. These show group averages ± SEM for the lateral forces exerted into the wall of the force channel. ***B***, Each panel shows data for trials with different target probability ratios, with traces within a panel grouped by different reward ratios.

**Figure 6.**
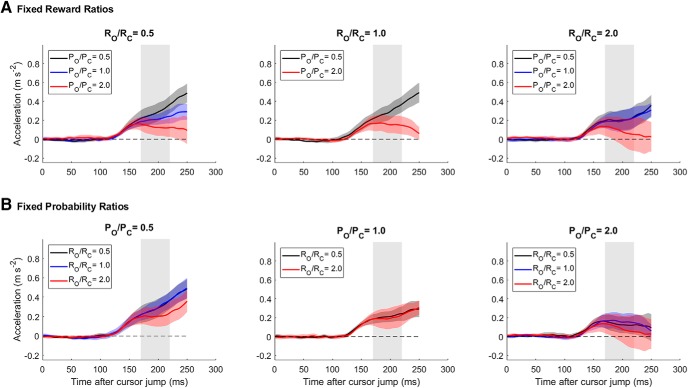
Acceleration responses to cursor jumps for all eight conditions in which the probabilities and rewards associated with center and outer targets were varied. ***A***, Each panel shows data for trials with different reward ratios, with traces within a panel grouped by different target probability ratios. These show group averages ± SEM for the lateral acceleration. ***B***, Each panel shows data for trials with different target probability ratios, with traces within a panel grouped by different reward ratios.

Figure 5 shows the average force traces from all eight conditions split in separate panels for the three reward ratios (Fig. 5*A*) and for the three target probability ratios (Fig. 5*B*). In general, the results show a tendency for greater corrective responses toward the center target for conditions in which the center target was more probable (Fig. 5*A*). The trend is less clear-cut for comparisons between conditions with different relative rewards, presumably due partly to the fact that the relative reward ratios were only 0.5, 1, and 2 in Experiment 3, rather than 0.05 and 20 in Experiment 1. The results for acceleration responses made in standard, nonchannel trials are very similar to the force responses in channel trials (Fig. 6).

We first examined the visuomotor response as a function of only the reward ratio or only the target probability ratio. The aim was to compare the modulatory effect of reward and probability on the visuomotor response with those observed in Experiments 1 and 2. Consistent with Experiments 1 and 2, regression analyses across conditions confirmed that the response size depended significantly on both variables separately (*p* < 10^−4^ and *p* = 0.009, respectively). Since the ratios of the experimental parameters varied nonlinearly across conditions in this experiment, we chose to use a log scale on which they are spaced linearly, thus facilitating model fitting and interpretation. Furthermore, neural recordings indicate that probabilistic information about which of two potential visual targets is correct on a given trial is encoded by neurons in a log probability ratio (Yang and Shadlen, 2007).

Given that both probability and reward significantly affected the visuomotor response gain individually, we examined how the gain modulation depended on the combination of the two in the form of expected value (the product of reward and probability). A rational agent, seeking to maximize cumulative reward, might modify the response gain according to log relative value, as follows:

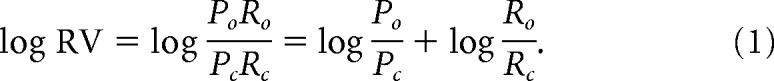


However, when comparing option values, which vary based on reward and probability, human decision-making tends not to be objectively rational according to expected value theory (Kahneman, 2003). In fact, humans can be risk seeking or risk averse in sensorimotor decision tasks (Nagengast et al., 2010, 2011a,b; Braun et al., 2011). That is, if participants are risk seeking, they may have a higher gain for the more rewarding target even if the probability of its specification is low. In contrast, risk-averse participants may always have a higher gain toward the more probable target even if it has lower reward. To model possible heterogeneity in risk sensitivity across subjects, a weighting parameter *w* was introduced which was restricted to take values between 0 and 1, as follows:

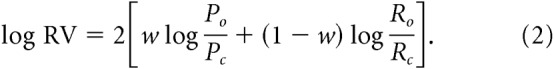


A value of *w* = 0.5 corresponds to rational risk-neutral behavior (as in Eq. 1), while a value *w* < 0.5 implies that a subject is risk seeking and is motivated more by the reward ratio. Finally, a value *w* > 0.5 implies risk aversion. It is also possible to interpret the *w* parameter as changing the relative value by an “intrinsic” reward (i.e., a reward associated with successfully reaching the target independent of the explicit experimenter-controlled reward; see Discussion).

**Figure 7.**
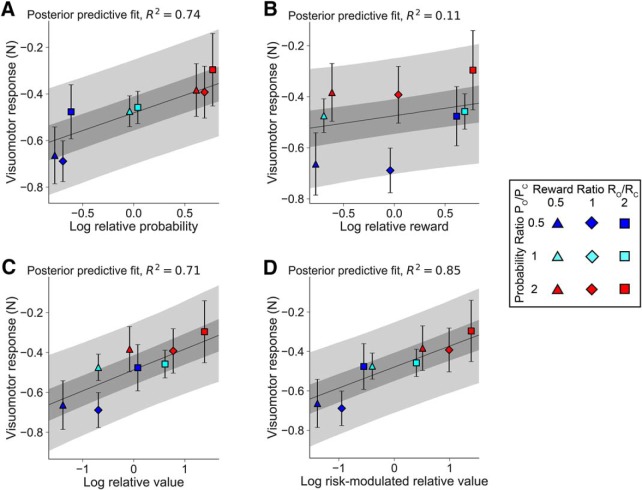
Visuomotor force responses in channel trials to varying both reward and probability ratios in Experiment 3. ***A–D***, Response size against log-relative probability (***A***), log-relative reward (***B***), log-relative value (***C***), and log risk-modulated relative value (***D***). The 50% and 95% credible regions are shaded in gray (darker and lighter regions, respectively).

We fit both the risk-neutral (Fig. 7*C*) and risk-sensitive (Fig. 7*D*) models. Given the sensitivity of model fitting to noise in the response gains, and the possible heterogeneity of risk attitudes across subjects, we fit the model parameters at both the individual subject-level and at the group-level via approximate Bayesian inference. This approach flexibly optimizes partial pooling across subjects at the group level, while adapting to subject-specific parameter differences (see Materials and Methods). We measured the quality of model fit using the Watanabe–Akaike information criterion (WAIC) as it is sensitive to the full posterior over the fitted parameters (as opposed to a point estimate as in other criterion measures) and has been shown to be more precise in estimating out-of-sample predictive accuracy (Vehtari et al., 2017). Model comparison showed that the risk-sensitive relative value model (WAIC = 7129) provided a better fit to the response gain data than any of the other models tested (risk-neutral relative value model WAIC = 7136; relative probability model WAIC = 7133; relative reward model WAIC = 7178). Estimating pairwise Bayes factors from WAIC scores results in factors of *K*_val_ = 33, *K*_prob_ = 7, *K*_rew_ > 150 when comparing the risk-neutral relative value, probability, and reward models, respectively, to the risk-sensitive relative value model. These Bayes factors indicate strong, positive, and very strong evidence in favor of the risk-sensitive relative value model, respectively (Kass and Raftery, 1995). With respect to the risk-sensitive relative value model, we examined risk sensitivity in visuomotor gain modulation by testing whether the weighting parameter in Equation 2 was significantly different from 0.5 (a weighting parameter of 0.5 would be equivalent to risk-neutral gain modulation as in Eq. 1). The weighting parameter *w* was estimated to be significantly >0.5 (mean, 0.68; 95% credible interval, 0.52–0.86) at the group level, indicating significant risk-aversion (or a nonzero contribution to value from a putative intrinsic reward associated with task success). Figure 7 shows the posterior predictive fits (i.e., the predicted visuomotor gains and estimation uncertainty) of the risk-neutral (Fig. 7*C*) and risk-sensitive (Fig. 7*D*) models with 50% and 95% credible regions in addition to probability (Fig. 7*A*) and reward (Fig. 7*B*) modulation models for comparison. The models were used to compute the mean decision parameter (e.g., risk-modulated log relative value in the case Fig. 7*D*) and response gain for each subject and unique experimental condition. The means across participants for each condition are plotted on top of the posterior predictive fits and the error bars reflect the SE. The risk-sensitive log relative value model explained the largest proportion (*R*^2^ = 0.85) of the response gain variance of any model.

For comparison with Experiments 1 and 2, we used ROC analysis to identify the time of first divergence in visuomotor responses between trials obtained in the blocks with most asymmetrical target values (*R_O_*/*R_C_* = 0.5, *P_O_*/*P_C_* = 0.5 versus *R_O_*/*R_C_* = 2, *P_O_*/*P_C_* = 2). The mean time of divergence was 151 ms for the asymmetrical value conditions, which was significantly later than the onset of the visuomotor response in a single-target condition (120 ms, paired *t* test, *t*_(9)_ = −2.3, *p* = 0.045).

**Figure 8.**
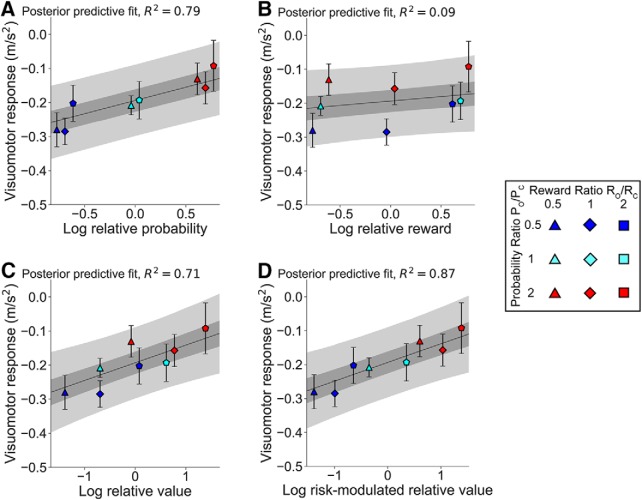
Visuomotor acceleration responses in standard trials to varying both reward and probability ratios in Experiment 3. ***A–D***, Response size against log-relative probability (***A***), log-relative reward (***B***), log-relative value (***C***), and log risk-modulated relative value (***D***). The 50% and 95% credible regions are shaded in gray (darker and lighter regions, respectively).

The analysis of hand acceleration responses in standard trials was very similar to the force responses (Fig. 6). Cross-condition regression analyses confirmed that the response size depended significantly on both probability and reward separately (*p* < 10^−5^ and *p* = 0.0008 respectively) and that the risk-sensitive relative value model (Fig. 8) explained the most variance in the acceleration data (*R*^2^ = 0.87).

**Figure 9.**
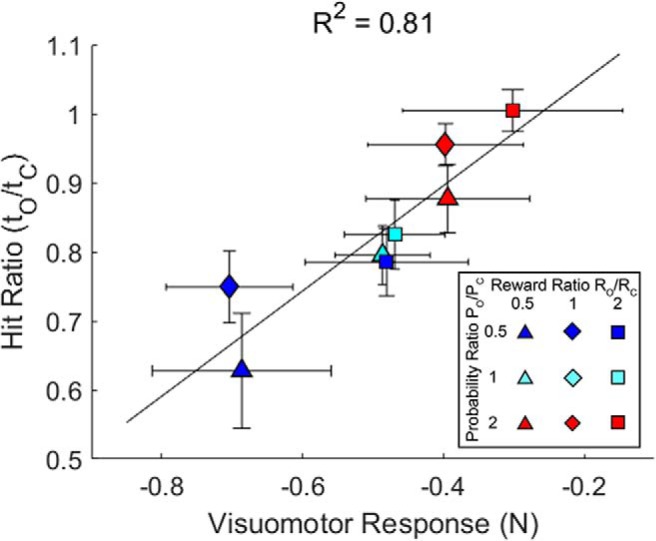
Target hit rate depends on the visuomotor response magnitude. The group average (±SEM) hit rate ratios are defined as the hit rate for outer targets (*t_O_*) divided by the hit rate for center targets (*t_C_*). These are plotted against the visuomotor response size (±SEM) for all combinations of relative reward and probability in Experiment 3. More negative visuomotor responses reflect greater correction of the cursor jump toward the center target. The line shows the linear regression fit.

Finally, we examined whether the ratio of hit rates for outer to center targets were related to the visuomotor response size across all reward and probability conditions (Fig. 9). Linear regression showed that there was a significant linear relationship (*p* = 0.002) between visuomotor response size and the relative proportions of outer and center target hits. Thus, rapid feedback response size was predictive of task success over a wide range of relative value conditions.

### Comparisons across experiments: task-dependent response modulation

There were differences in task features among the three experiments that comprise this study, as well as differences in absolute visuomotor response magnitudes (especially between Experiments 1 and 2 vs Experiment 3). It seems highly likely that the overall task context (e.g., how many potential targets were available, and the proportion of channel trials and standard trials with a cursor jump perturbation set for each target) was a key factor that mediated response magnitude across conditions and experiments, independently of value manipulations. For example, the visuomotor response magnitudes when the center target was more rewarded or more probable in Experiments 1 and 2 were smaller than those observed in the single-target condition, despite a 20-fold larger reward, or 2-fold greater probability, for the center target. By contrast, response magnitudes when the relative value for the center target was greatest in Experiment 3 were comparable to the single-target condition. This general notion of task-dependent reflex modulation is consistent with a large body of published work on reflex function (Hammond, 1956), whereas our article is focused on the influence of high and low value targets on visuomotor responses under identical task conditions in each experiment. The specific task constraints we used to interrogate the effects of value manipulations in each experiment almost certainly influenced the absolute response magnitudes. However, the influence of value on the relative response magnitudes was consistent across experiments despite the different task features. Therefore, the effects of value we describe are unlikely to depend strongly on the particular task configuration.

## Discussion

Our results show that the size of rapid visuomotor responses to a sudden change in visual feedback about limb position depends upon the relative values of potential movement goals. Previous work showed that rapid feedback responses are flexibly regulated according to multiple components of the task, including timing (Franklin and Wolpert, 2008; Cluff and Scott, 2015) and spatial precision constraints (Gallivan et al., 2016), the presence of obstacles in the environment (Nashed et al., 2012, 2014), and the energetic or control costs associated with potential corrective responses (Nashed et al., 2012, 2014; Pruszynski et al., 2014). Rapid feedback responses can also reflect a control policy intermediate between those associated with competing goals when there is uncertainty about which goal will ultimately be specified (Gallivan et al., 2016). This illustrates that sensorimotor control policies are sufficiently flexible to take account of multiple potential goals simultaneously. However, this previous work that examined the flexibility of fast feedback responses manipulated physical characteristics of the task that are inherently coupled to the required motor outputs. Our current results show that feedback control is sensitive to decision variables, such as prospective reward, under otherwise identical task conditions. This suggests that feedback control policies that govern state-dependent transformations of sensory feedback to motor commands can be tailored to implement value-based choice.

Previous work showed that the magnitude rapid feedback responses to target perturbations or rapid target onsets was little affected by asymmetrical presentation probability (Reynolds and Day, 2012; Pruszynski et al., 2016). We see two potential explanations for this discrepancy with our current findings about visuomotor responses to perturbations of hand position feedback. One possibility is that automatic responses to target jumps are insensitive to value asymmetry, in contrast to responses to our current results for cursor jumps. These different forms of perturbation appear to involve dissociable response processes (Franklin et al., 2016; for review, see Oostwoud Wijdenes and Medendorp, 2017). An alternative possibility, which we favor, is that automatic feedback responses are more strongly modulated on the basis of expected value when reflex tuning is necessary to achieve task success. Pilot experiments for this study suggested that the effects of reward on visuomotor responses were negligible when subjects could hit the target on every trial. This situation of assured success appears to be present in both the Reynolds and Day (2012) and Pruszynski et al. (2016) studies, neither of which enforced a time deadline for target acquisition. Nonetheless, it is important to note that our conclusions regarding the capacity for rapid feedback corrections to be modulated according to expected value may hold specifically for visual perturbations of limb position feedback under conditions of time pressure. More generally, while our laboratory task shows that value can affect reflexes under controlled conditions, the extent and importance of such modulation in real-world tasks is still an open question.

Our results also extend previous findings that evolving decisions and reward or action history can bias the state of the motor system toward favored potential actions during motor planning (Platt and Glimcher, 1999; Dorris et al., 2000; Lauwereyns et al., 2002; Milstein and Dorris, 2007; Marinovic et al., 2017). This previous work showed that neural activity in the saccadic and reach control networks is increased for neurons encoding high-value actions, and leads to biases in motor behavior toward favored actions. In our task, such neural activity cannot be biased in favor of a specific action (e.g., leftward or rightward hand force) as a function of reward or an ongoing decision, but rather decision variables must flexibly modulate the magnitude and direction of hand force as a function of evolving sensory input (i.e., depending on the direction of the cursor jump). The fast visuomotor response cannot therefore involve the relatively slow evaluation of evidence in higher-order brain areas traditionally associated with cognitive decision-making. This does not imply that high-order cognitive areas are not critical for making value estimations, or even for setting reflex gains, before movement, to reflect trial-by-trial updates to value-based decision processes. Indeed, Franklin et al. (2014) showed that the brain can learn different visuomotor response gains for left and right cursor perturbations depending on whether or not the perturbations in each direction were task relevant. This work showed that visuomotor responses can be modulated appropriately to hit targets when distinct perturbation types can be predicted based on the direction of initial cursor deviation. In the current study, we show that visuomotor responses are modified for identical perturbations as a function of target value. Thus, it appears that low-level sensorimotor areas initiate motor decisions on the basis of the expected values of response alternatives, using the continuous stream of sensory evidence available during an individual movement. In this sense, fast visuomotor responses make a contribution to value-based decision-making.

If low-level sensorimotor circuits can contribute to value-based decisions through continuous feedback control, rather than merely executing the outcome of discrete action decisions taken in higher-order brain areas, it would support for the hypothesis that value-based decision algorithms are distributed throughout multiple levels of sensorimotor and cognitive processing hierarchies (Hunt et al., 2014; Hunt and Hayden, 2017). This notion differs from the traditional view that decisions arise from a serial process with modular units for choice evaluation, value comparison and action selection. According to the alternative view, the basis for decisions is mutual inhibition between neural representations of alternative options, and these computations occur simultaneously in multiple brain areas along both motor and abstract-value dimensions of tasks (Wang, 2012). Our current evidence that value-based decisions can be implemented through sensorimotor feedback control supports the alternative view, and the general notion that behavior emerges via a distributed consensus between circuits engaged nominally in decision and sensorimotor processes (Cisek, 2012).

The regulation of rapid feedback responses was associated with successful pursuit of high-value task outcomes in our experiments, suggesting that value-based response tuning is functionally advantageous. This makes ethological sense in a dynamic world in which information about response options can change rapidly. For example, if a movement is perturbed such that an action associated with high reward becomes available, feedback corrections that oppose the perturbation should be inhibited to maximize the rewards obtained. This resembles a minimum intervention principle, in which errors are only corrected if they directly interfere with attainment of the task goal, and which is a hallmark of optimal feedback control systems (Todorov and Jordan, 2002; Diedrichsen, 2007; Liu and Todorov, 2007). In our task, when the cursor jumped to one side, the new lateral position of the cursor was often in a better state to maximize value than the unperturbed position, and so the jump was little corrected or even exaggerated if outer targets were much more valuable than the center target. Thus, feedback control cannot only reduce the costs associated with achieving a particular outcome, but at a broader level implement policies that include decision processes that evaluate and select goals (Nagengast et al., 2010; Braun et al., 2011; Wolpert and Landy, 2012; Christopoulos and Schrater, 2015).

A rational agent seeking to maximize cumulative rewards in the long run should make choices according to the relative value of available options. However, humans and other animals often behave according to risk-modulated value functions; they make choices that lead to lower overall gains by favoring larger, less certain rewards when risk seeking, or more certain but smaller rewards when risk averse. Both risk-seeking and risk-averse behavior have been exhibited in motor decision tasks, depending on factors such as the probability of successful outcomes (Trommershäuser et al., 2008; Wu et al., 2009; Nagengast et al., 2010, 2011b; McDougle et al., 2016). Interestingly, the valence of risk modulation in motor tasks is often mirror opposite to that observed for economic decisions (Wu et al., 2009; McDougle et al., 2016). Our current results suggest that rapid feedback responses are tuned to a risk-averse value function. An interesting future question might be to determine whether an individual's risk sensitivity in visuomotor response regulation correlates with their risk sensitivity in cognitive decision-making.

An alternative perspective on the apparent risk-aversion evident for motor decisions in this study is that there is an “intrinsic” reward associated with successfully attaining a motor goal. Indeed, in Experiment 2, rapid visuomotor responses and task performance were biased toward more probable targets in the absence of any financial reward. If these results are to be interpreted in a value-based decision-making framework, then a nonzero intrinsic reward component to value is obligatory. More broadly, the fact that humans sometimes decide to perform costly and difficult movements in the absence of explicit rewards, for example in (nonprofessional) sport and the performance arts, suggests a capacity to arbitrarily assign intrinsic value to completion of challenging physical tasks. Such flexibility in value assignment might be necessary to afford humans the capacity to make decisions based on complex reasoning or affect, rather than simply on the prospect of explicit reward. An important corollary of this idea is that if the attainment of intrinsic rewards is a general feature of successful completion of goal-directed sensorimotor tasks, experimental manipulation of reward through payment of money or provision of food would tend to underestimate the composite value derived from any given action. This scenario would complicate interpretations of choice behavior involving motor tasks based on prospect theory. Nonetheless, our results clearly show that feedback control policies are biased to favor more frequent task success at the expense of the overall extrinsic reward accumulated through movement. This implies that low-level visuomotor feedback loops can reflect the outcomes of nuanced choice algorithms associated with value based decision-making.
